# Temporal changes in cause‐specific death in men with localised prostate cancer treated with radical prostatectomy: a population‐based, nationwide study

**DOI:** 10.1002/jso.26579

**Published:** 2021-06-18

**Authors:** Frederik B. Thomsen, Hans Garmo, Klaus Brasso, Lars Egevad, Pär Stattin

**Affiliations:** ^1^ Department of Urology, Copenhagen University Hospital – Rigshospitalet Copenhagen Prostate Cancer Center Copenhagen Denmark; ^2^ Department of Clinical Medicine University of Copenhagen Copenhagen Denmark; ^3^ Regional Cancer Centre Uppsala Örebro Uppsala University Hospital Uppsala Sweden; ^4^ Division of Cancer Studies, King's College London, School of Medicine Cancer Epidemiology Group London UK; ^5^ Department of Oncology‐Pathology, Karolinska Institutet Karolinska University Hospital, Solna Stockholm Sweden; ^6^ Department of Surgical Sciences Uppsala University Hospital Uppsala Sweden; ^7^ Department of Surgical and Perioperative Sciences, Urology, and Andrology Umeå University Hospital Umeå Sweden

**Keywords:** mortality, PCBaSe, prostate cancer, radical prostatectomy

## Abstract

**Background and Objective:**

Changes in diagnostic work‐up, histopathological assessment, and treatment of men with prostate cancer during the last 20 years have affected the prognosis. The objective was to investigate the risk of prostate cancer death in men with clinically localised prostate cancer treated with radical prostatectomy in Sweden in 2000–2010.

**Methods:**

Population‐based, nationwide, study on men with clinically localised prostate cancer treated with radical prostatectomy in the period 2000–2010. Cox regression analyses were used to assess differences in risk of prostate cancer death according to calendar period for diagnosis and stratified on risk category.

**Results:**

The study included 19 330 men with a median follow‐up of 12.4 years. Men diagnosed in 2007–2008 and 2009–2010 had a significantly lower risk of prostate cancer death compared to men diagnosed in 2000–2002. The reduced risk of prostate cancer death was restricted to men with intermediate‐risk prostate cancer with no differences observed in men with low‐ or high‐risk prostate cancer.

**Conclusion:**

During the study period, the risk of prostate cancer death decreased in the total population of men with localised prostate cancer treated with radical prostatectomy. The decrease was restricted to men with intermediate‐risk prostate cancer.

## 1. INTRODUCTION

To inform on treatment options knowledge of the natural history of the disease at hand as well as the effect of interventions are imperative. The natural history of localised prostate cancer is best known in men diagnosed in the 1970s and 1980s,^1^, ^2^ while outcomes following curatively intended treatment have primarily been studied in randomised controlled trials in men diagnosed in the 1990s and 2000s.^3^, ^4^, ^5^, ^6^

During the 1990s and 2000s an increased use of prostate specific‐antigen (PSA) testing, increased number of diagnostic biopsies sampled, and revisions of histopathological grading criteria occurred.^7^, ^8^, ^9^, ^10^ Taken together these changes have caused a stage and grade migration,^11^, ^12^, ^13^, ^14^ which resulted in a seemingly better prognosis for men with prostate cancer diagnosed in more contemporary calendar periods.^15^, ^16^

The aim of the present study was to investigate temporal changes in the risk of prostate cancer death in men with localised prostate cancer treated with radical prostatectomy in Sweden in 2000–2010.

## 2. MATERIAL AND METHODS

Prostate Cancer data Base Sweden (PCBaSe) 4.0 contains information on cancer characteristics and primary treatment from the National Prostate Cancer Register (NPCR).^17^, ^18^ Data on comorbidity (CCI) were obtained from the Patient Registry, data on educational level and marital status were obtained from The Longitudinal database on socioeconomic factors (LISA), and data on cause and date of death were obtained from the Cause of Death Registry.^18^, ^19^, ^20^, ^21^, ^22^, ^23^

This study included men with clinically localised prostate cancer aged 75 years or younger diagnosed in the period 2000–2010 who underwent radical prostatectomy within 6 months from the date of diagnosis. Localised prostate cancer was defined as: T1‐2, PSA < 50 ng/ml and without evidence of dissemination (N0/x, M0/x). We used a modification of the NCCN risk categorisation to stratify on risk categories: Low‐risk (T1‐2 and PSA < 10 ng/ml and Gleason score ≤ 6); intermediate‐risk (T1‐2 and/or PSA 10–20 ng/ml and/or Gleason score 7 [3 + 4 or 4 + 3]), and high‐risk (T1‐2 and/or PSA > 20 ng/ml and/or Gleason score 8–10).

The following variables were available: age at diagnosis, T stage, Gleason score on biopsy, serum level of PSA (ng/ml), the proportion of positive biopsy cores, CCI, education level, marital status, and the annual number of radical prostatectomies performed at treating hospital the year before diagnosis.

Follow‐up was calculated from the date of prostate cancer diagnosis to death, emigration, or end of the study period (December 31, 2019), whichever came first. Death was classified as prostate cancer death or non‐prostate cancer death. The Research Ethics Board at Umeå University Hospital approved the study.

### 2.1. Statistical methods

Follow‐up was calculated with the reverse Kaplan–Meier method. Cumulative incidences with 95% confidence intervals (CI) of prostate cancer death were estimated with competing risk analyses treating death from other causes as competing events and vice versa. Uni‐ and multivariable Cox regression analyses were applied to investigate the association between risk of prostate cancer death by year of diagnosis (2000–2002, 2003–2004, 2005–2006, 2007–2008, and 2009–2010), age (<60, 60–<65, 65–<70, 70+), T stage (1, 2), Gleason score (≤6, 7 (3 + 4), 7 (4 + 3), 8, and 9–10), PSA (0–<10, 10–<20, and 20–<50), the proportion of positive biopsy cores (<16.7%, 16.7%–33%, 34%–50%, >50%, and missing), CCI (0, 1, and 2+), education level (low, middle, high, and missing), marital status (married and not married), and the annual number of radical prostatectomies performed at treating hospital the year before treatment (stratified in quartiles).

All tests were two‐sided and the significance level was set to *p* < 0.05. Statistical analysis was performed with R version 4.0.0 (R Foundation for Statistical Computing).

## 3. RESULTS

The study includes 19 330 men diagnosed with localised prostate cancer in 2000–2010 who underwent primary radical prostatectomy within 6 months from diagnosis. The median follow‐up was 12.4 years, ranging from 9.1 to 17.2 years. During the study period the proportion of men aged 65 years or above increased, as did the proportion of men with clinical stage T1 and men with Gleason scores ≤ 6, Table 1. The number of biopsy cores increased from a median of 6 (IQR 6–6) in 2000–2002 to 10 (IQR 8–10) in 2009–2010. Baseline characteristics stratified on risk category are available in Table S1. In men with low‐risk prostate cancer there was an increase in the proportion of men with T1 tumours. In men with intermediate‐ and high‐risk prostate cancer the median PSA decreased while there was an increase in proportion of men with T1 tumours. In men with high‐risk prostate cancer, the proportion of men with Gleason scores 8–10 increased from 53% in 2000–2002 to 67% in 2009–2010 and the proportion of men with CCI 1 or higher increased from 8% in 2000–2002 to 17% in 2009–2010.

**Table 1:** Baseline characteristics of 19 330 men in Prostate Cancer data Base Sweden (PCBaSe) diagnosed with localised prostate cancer and treated with radical prostatectomy stratified on calendar period of diagnosis

	2000–2002	2003–2004	2005–2006	2007–2008	2009–2010
	*n* = 2544	*n* = 3353	*n* = 3743	*n* = 3605	*n* = 2298
Age at diagnosis, years					
<60	984 (35)	1202 (32)	1268 (31)	1171 (30)	1316 (28)
60–<65	886 (31)	1235 (33)	1441 (35)	1325 (34)	1480 (31)
65–<70	766 (27)	973 (26)	1050 (26)	1120 (28)	1462 (31)
70+	191 (7)	297 (8)	350 (9)	315 (8)	498 (10)
Clinical tumour category					
T1	1650 (58)	2366 (64)	2763 (67)	2570 (65)	3223 (68)
T2	1177 (42)	1341 (36)	1346 (33)	1361 (35)	1533 (32)
Gleason score					
≤6	1986 (76)	2589 (71)	2701 (67)	2224 (57)	2354 (50)
7 (3 + 4)	359 (14)	683 (19)	805 (20)	1055 (27)	1465 (31)
7 (4 + 3)	136 (5)	221 (6)	326 (8)	380 (10)	572 (12)
8	97 (4)	137 (4)	167 (4)	203 (5)	276 (6)
9–10	29 (1)	31 (1)	39 (1)	51 (1)	78 (2)
PSA at diagnosis, ng/ml					
Median (IQR)	8 (5–11)	7 (5–11)	7 (5–10)	7 (5–10)	6 (5–10)
Proportion positive biopsy cores, %					
Median (IQR)	33 (17–50)	33 (17–50)	33 (17–50)	33 (20–50)	30 (20–50)
Missing	1 749 (69%)	304 (9%)	406 (11%)	113 (3%)	74 (3%)
Charlson comorbidity index					
0	2588 (92)	3319 (90)	3621 (88)	3443 (88)	4106 (86)
1	166 (6)	285 (8)	350 (9)	359 (9)	470 (10)
2+	73 (3)	103 (3)	138 (3)	129 (3)	180 (4)
Education level					
Low	896 (32)	1102 (30)	1145 (28)	1054 (27)	1226 (26)
Middle	1143 (40)	1524 (41)	1698 (41)	1615 (41)	1949 (41)
High	776 (27)	1066 (29)	1251 (30)	1244 (32)	1564 (33)
Missing	12 (0)	15 (0)	15 (0)	18 (0)	17 (0)
Marital status					
Married	2179 (77)	2779 (75)	3066 (75)	2877 (73)	3367 (71)
Not married	648 (23)	928 (25)	1043 (25)	1054 (27)	1389 (29)
Annual procedures performed at treating hospital					
Median (IQR)	38 (19–74)	53 (29–121)	62 (40–126)	60 (37–131)	69 (40–‐159)

Abbreviations: IQR, interquartile range; PSA, prostate specific‐antigen.

**Figure 1:**
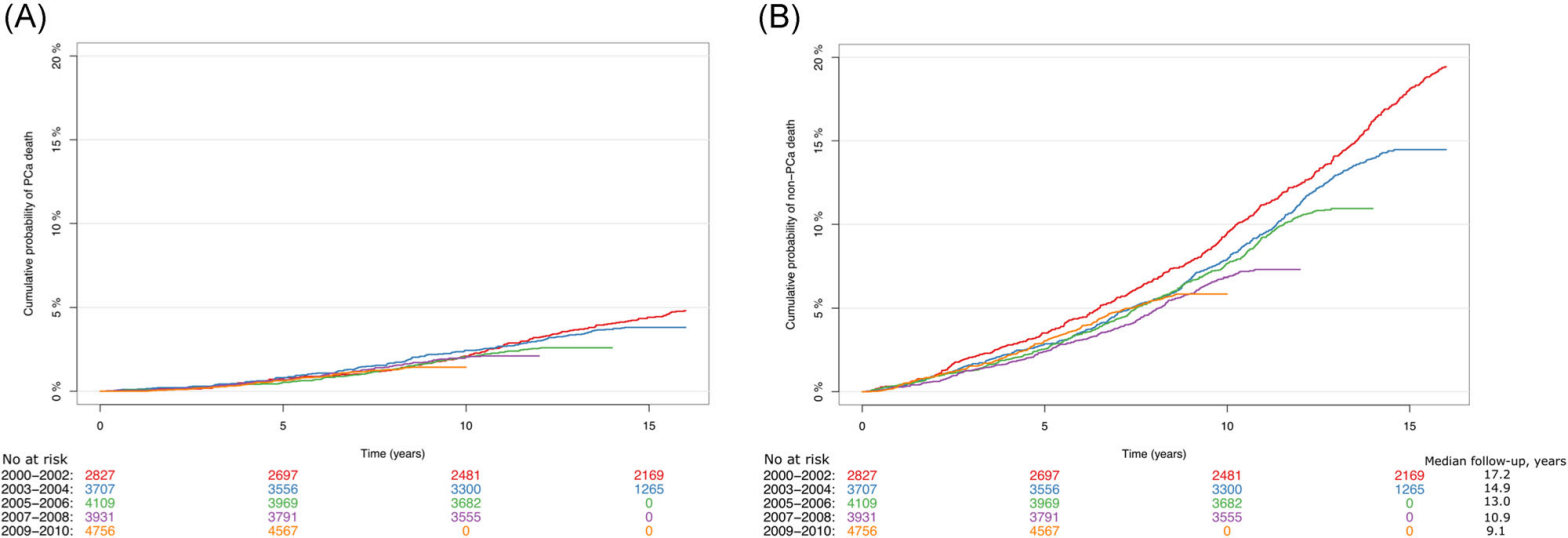
Cumulative probability of (A) prostate cancer (PCa) and (B) non‐PCa deaths for men with localised PCa treated with radical prostatectomy in 2000–2010 stratified on diagnostic period [Color figure can be viewed at wileyonlinelibrary.com]

The 9‐year overall mortality decreased from 9.4% (95% CI 8.4%–10.5%) in 2000–2002 to 7.3% (95% CI 6.5%–8%) in 2009–2010. The cumulative incidence of prostate cancer and nonprostate cancer death is depicted in Figure 1. Overall, cumulative prostate cancer deaths following 9 years remained unchanged with 1.7% (95% CI 1.2%–2.1%) of men diagnosed in 2000–2002 compared with 1.4% (95% CI 1.1%–1.8%) of men in 2009–2010. In the same period, the nonprostate cancer deaths declined from 7.8% (95% CI 6.8%–8.8%) to 5.9% (95% CI 5.2%–6.5%). In univariable Cox regression analyses lower risk of prostate cancer death was seen in men diagnosed in later calendar periods, men with higher education level and men diagnosed in hospitals performing the highest annual number of prostatectomies, Table 2; whereas the higher risk of prostate cancer death was seen in older men, men with T2, higher Gleason score, higher PSA, more cancer in biopsies and CCI 2 or higher. In multivariate Cox regression analyses, men diagnosed in 2007–2008 (hazard ratio [HR] 0.70 [95% CI 0.49–0.97]) and 2009–2010 (HR 0.58 [95% CI 0.41–0.84]) had a lower risk of prostate cancer death compared with men diagnosed in 2000–2002; whereas age, T stage, Gleason score, PSA, cancer in biopsies, and comorbidity still were associated with a higher risk of prostate cancer death.

**Table 2:** Uni‐ and multivariable Cox regression analyses for risk of prostate cancer death

		Univariable		Multivariable	
		HR	95% CI	HR	95% CI
Diagnosis period					
	2000–2002	Ref		Ref	
	2003–2004	0.84	(0.66–1.07)	1.06	(0.80–1.42)
	2005–2006	0.65	(0.50–0.85)	0.80	(0.59–1.09)
	2007–2008	0.66	(0.49–0.87)	0.70	(0.49–0.97)
	2009–2010	0.59	(0.43–0.80)	0.58	(0.41–0.84)
Age, year					
	<60	Ref		Ref	
	60–<65	1.29	(1.02–1.64)	1.16	(0.91–1.49)
	65–<70	1.90	(1.51–2.40)	1.54	(1.21–1.97)
	70+	2.85	(2.14–3.79)	1.77	(1.31–2.39)
Clinical tumour category					
	T1	Ref		Ref	
	T2	2.64	(2.22–3.13)	1.65	(1.37–1.98)
Gleason score					
	≤6	Ref		Ref	
	7 (3 + 4)	2.93	(2.33–3.69)	2.43	(1.92–3.09)
	7 (4 + 3)	5.28	(4.06–6.86)	4.11	(3.14–5.38)
	8	7.67	(5.79–10.16)	6.16	(4.61–8.23)
	9–10	25.03	(18.27–34.25)	16.76	(12.06–23.30)
PSA, ng/ml					
	0–10	Ref		Ref	
	10–20	2.09	(1.74–2.51)	1.39	(1.14–1.70)
	20–50	3.48	(2.64–4.57)	2.09	(1.57–2.78)
Proportion positive biopsy core					
	<16.7%	Ref		Ref	
	16.7%–33%	1.24	(0.81–1.90)	1.35	(0.87–2.11)
	34%–50%	2.61	(1.88–3.62)	2.20	(1.56–3.10)
	>50%	5.06	(3.62–7.04)	3.53	(2.48–5.02)
	Missing	3.15	(2.23–4.45)	2.39	(1.61–3.55)
Charlson comorbidity index					
	0	Ref		Ref	
	1	1.16	(0.86–1.56)	1.01	(0.73–1.38)
	2+	1.93	(1.29–2.88)	1.90	(1.25–2.87)
Education					
	Low	Ref		Ref	
	Middle	0.81	(0.67–0.99)	0.92	(0.75–1.14)
	High	0.79	(0.64–0.99)	0.94	(0.75–1.18)
	Missing	1.41	(0.45–4.42)	0.81	(0.20–3.25)
Marital status					
	Married	Ref		Ref	
	Not married	1.11	(0.91–1.37)	1.17	(0.96–1.42)
No. procedures performed at treating hospital					
	1st Quartile	Ref		Ref	
	2nd Quartile	1.03	(0.85–1.26)	1.06	(0.86–1.31)
	3rd Quartile	0.67	(0.82–1.18)	1.27	(0.83–1.95)
	4th Quartile	0.67	(0.50–0.84)	0.76	(0.58–1.01)

Abbreviations: CI, confidence interval; HR, hazard ratio; PSA prostate specific‐antigen.

**Figure 2:**
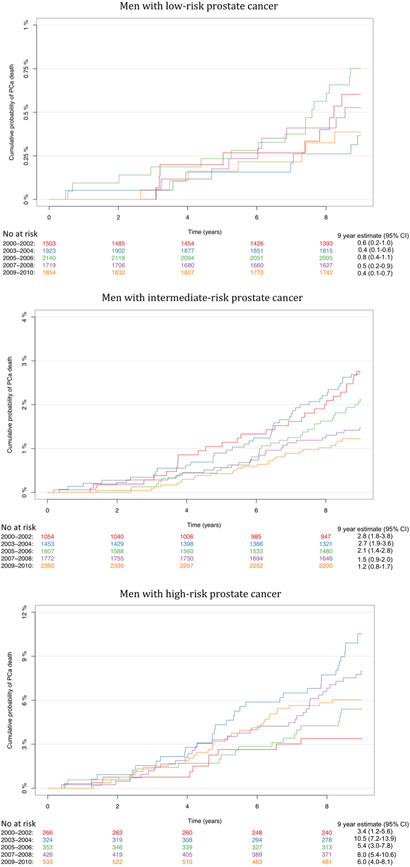
Cumulative probability of prostate cancer (PCa) deaths for men with localised PCa treated with radical prostatectomy in 2000–2010 stratified on diagnostic period and risk group [Color figure can be viewed at wileyonlinelibrary.com]

Stratified on risk category the cumulative prostate cancer mortality was 0.6% (95% CI 0.2%–1.0%) in 2000–2002 and 0.4% (95% CI 0.1%–0.7%) in 2009–2010 for men with low‐risk prostate cancer, Figure 2. Corresponding numbers for men with intermediate‐risk and high‐risk prostate cancer were 2.8% (95% CI 1.8%–3.8%) and 1.2% (95% CI 0.8%–1.7%), and 3.4% (1.2–5.6) and 6.0% (95% CI 4.0%–8.1%), respectively. In men with intermediate‐risk prostate cancer the cumulative prostate cancer deaths decreased significantly by calendar period for diagnosis, whereas cumulative prostate cancer death showed no systematic change over time in men with low‐ or high‐risk prostate cancer, Table 3. In addition, the ratio of prostate cancer deaths/nonprostate cancer deaths decreased in men with intermediate‐risk prostate cancer from 0.24 in 2000–2002 to 0.15 in 2009–2010.

**Table 3:** Number of prostate cancer and nonprostate cancer deaths with 9 years follow‐up and 9 year cumulative incidence estimates

	Prostate cancer death		Nonprostate cancer death			
	No of events/no at risk	Cumulative incidence% (95% CI)	No of events/no at risk	Cumulative incidence% (95% CI)	No prostate cancer events/no of deaths from any cause	Prostate cancer mortality ratio
Low‐risk						
2000–2002	9/1 503	0.6 (0.2–1)	108/1 503	7.2 (5.9–8.5)	9/117	0.08
2003–2004	7/1 923	0.4 (0.1–0.6)	109/1 923	5.7 (4.7–6.7)	7/116	0.06
2005–2006	16/2 140	0.8 (0.4–1.1)	128/2 140	6.0 (5.0–7.0)	16/144	0.11
2007––2008	9/1 719	0.5 (0.2–0.9)	88/1 719	5.2 (4.1–6.2)	9/97	0.09
2009–2010	7/1 854	0.4 (0.1–0.7)	103/1 854	5.6 (4.6–6.7)	7/110	0.06
Intermediate‐risk						
2000–2002	29/1 054	2.8 (1.8–3.8)	92/1 054	8.8 (7.1–10.5)	29/121	0.24
2003–2004	40/1 453	2.8 (1.9–3.6)	118/1 453	8.1 (6.7–9.6)	40/158	0.25
2005–2006	34/1 607	2.1 (1.4–2.8)	110/1 607	6.9 (5.6–8.1)	34/144	0.24
2007–2008	26/1 772	1.5 (0.9–2.0)	112/1 772	6.3 (5.2–7.5)	26/138	0.19
2009–2010	28/2 360	1.2 (0.8–1.7)	158/2 360	5.6 (4.7–6.5)	28/186	0.15
High‐risk						
2000–2002	9/266	3.4 (1.2–5.6)	19/266	7.2 (4.1–10.3)	9/28	0.32
2003––2004	34/324	10.5 (7.2–13.9)	21/324	6.5 (3.8–9.2)	34/55	0.62
2005–2006	19/353	5.4 (3.0–7.8)	32/353	9.1 (6.1–12.1)	19/51	0.37
2007–2008	34/426	8.0 (5.4–10.6)	28/426	6.6 (4.2–8.9)	34/62	0.55
2009–2010	32/533	6.0 (4.0–8.1)	40/533	7.6 (5.3–9.9)	32/72	0.44

Abbreviation: CI, confidence interval.

In multivariable Cox regression analyses men with intermediate‐risk prostate cancer diagnosed in 2007–2008 (HR 0.56 [95% CI 0.33–0.95]) and 2009–2010 (HR 0.47 [95% CI 0.27–0.82]) had a significantly lower risk of prostate cancer death compared with men diagnosed in 2000–2002, Table 4. There was no significant difference in risk of prostate cancer death between diagnostic periods for men classified with low‐ or high‐risk prostate cancer. Full multivariable Cox regression analyses stratified on risk category are available in Table S2.

**Table 4:** Multivariate Cox regression analyses for risk of death from prostate cancer stratified on risk category

		Low‐risk		Intermediate‐risk		High‐risk	
		HR	95% CI	HR	95% CI	HR	95% CI
Diagnostic period							
	2000–2002	Ref		Ref		Ref	
	2003–2004	0.61	(0.32–1.16)	1.10	(0.72–1.69)	1.36	(0.83–2.24)
	2005–2006	0.64	(0.33–1.27)	0.86	(0.55–1.35)	0.79	(0.45–1.39)
	2007–2008	0.63	(0.28–1.42)	0.56	(0.33–0.95)	0.98	(0.56–1.69)
	2009–2010	0.52	(0.20–1.34)	0.47	(0.27–0.82)	0.80	(0.45–1.42)

*Note*: Adjusted for age at diagnosis, clinical tumour category, Gleason score, prostate‐specific antigen, percent positive biopsy cores, CCI, education, marital status, and a number of procedures performed at treating hospital.

Abbreviations: CI, confidence interval; HR, hazard ratio.

## 4. DISCUSSION

In this nationwide, population‐based study of Swedish men diagnosed with localised prostate cancer in 2000–2010 treated with primary radical prostatectomy, we found lower all‐cause mortality in men diagnosed in more recent calendar periods, which was caused by a decrease in nonprostate cancer deaths. This is likely the result of an increased life expectancy in the general Swedish population.^24^ Only in men with intermediate‐risk prostate cancer was there a decrease in risk of prostate cancer death, whereas no significant changes were found in men with low‐ or high‐risk prostate cancer.

Limitations of this study include the use of data from the original histopathological reports of diagnostic prostate biopsies without centralised review and that we were unable to assess the impact on the outcome of each change that occurred during the study period. Strengths of our study include a large nationwide, population‐based cohort of men with comprehensive data from several high‐quality healthcare registers.^18^, ^19^, ^20^ We chose to only include men treated with radical prostatectomy to try and reduce biases caused by changes in treatment strategies. Besides changes in surgical technique, changes in case‐mix, work‐up, and histopathological assessment of biopsies likely also affected outcome. During the study period, an increasing proportion of men with very low‐risk prostate cancer were managed by active surveillance^25^ and more men with high‐risk prostate cancer and high comorbidity underwent radical prostatectomy (Table S1). This could be the reason why we did not detect any changes in the risk of prostate cancer death in these risk categories.

A number of changes occurred during the study period, which may have contributed to the fewer number of deaths in men diagnosed with intermediate‐risk prostate cancer in the most recent calendar periods. Because of the increased use of PSA testing in asymptomatic men, the incidence of prostate cancer has drastically increased during the last decades in most Western countries.^26^, ^27^ PSA testing in asymptomatic men effectively changes the stage at diagnosis by significantly reducing the number of men diagnosed with advanced prostate cancer and consequently increases the number of men diagnosed with localised prostate cancer.^28^, ^29^ Additionally, the median number of biopsy cores taken at diagnostic work‐up increased from 6 to 10, which results in an estimated increase in the number of prostate cancers detected by 31%, including small, clinically insignificant cancers.^9^ Finally, the Gleason grading guidelines have changed, which has caused a grade migration upwards. Originally the Gleason score of needle biopsies was calculated as “the sum of the two predominant grades,”^30^ which in the modified 2005 guidelines were changed to “the sum of the most predominant grade and the highest grade present.”^10^ In cases where the predominant grades included the highest grade present, lower grades should only be included in the Gleason score if they constituted more than 5% of the tumour volume. Moreover, regular cribriform glands, which were previously classified as Gleason pattern 3 were now considered Gleason pattern 4. In Sweden among men diagnosed with stage T1 prostate cancer and PSA 4–10 ng/ml the proportion of men with Gleason scores 7–10 in the diagnostic biopsy specimen increased from 18% in 1998 to 40% in 2011.^11^ Other studies have shown that 34%–55% men with original Gleason score 6 were reclassified with higher Gleason scores when assessed according to the modified 2005 guidelines.^31^, ^32^, ^33^ Thus, without change in the overall prognosis, the prognosis of both Gleason score 6 and Gleason score 7 (3 + 4) improved, as the most aggressive prostate cancers that used to be assigned a Gleason score 6 would be reclassified as Gleason score 7 (3 + 4), that is, an example of Will Rogers phenomenon. Thus, the fewer observed prostate cancer deaths in men with intermediate‐risk prostate cancer seem logical because the influence of the stage and grade migration influenced outcome for this risk group the most.

Risk stratification of men with localised prostate cancer is based on T stage, Gleason score, and PSA. The original D'Amico risk classification stratified men into three risk groups: low‐, intermediate‐, and high‐risk.^34^ In recent years “very low‐risk prostate cancer” has been included as a fourth risk group accounting also for the prostate volume.^35^ The original D'Amico definition of intermediate‐risk prostate cancer was based on three intermediate‐risk criteria: T2b, PSA 10–20 ng/ml, and Gleason score 7.^34^ Thus, men can have from 1 to 3 intermediate‐risk criteria. The risk of biochemical recurrence following radical prostatectomy is higher in men with 2–3 intermediate‐risk criteria compared with men with only 1 intermediate‐risk criterion.^36^ This suggests that men with intermediate‐risk prostate cancer could likely be stratified in a “favourable” and an “unfavourable” risk group.

The lower prostate cancer mortality indicates that active surveillance could be an alternative strategy for some men with intermediate‐risk prostate cancer. To date no randomised study has compared radical prostatectomy to an active surveillance strategy. But a recent matched‐pair analysis of two nationwide Danish cohorts found no difference in prostate cancer mortality after 10 years between 276 men with intermediate‐risk prostate cancer treated with radical prostatectomy and 271 men with intermediate‐risk prostate cancer managed on active surveillance in 2002–2012.^37^ The majority of men in this study had one intermediate‐risk criterion and either Gleason score 6 or 7 (3 + 4). We still need additional studies to investigate this strategies safety in these men.

In the 2010s MRI has been introduced in the diagnostic work‐up of prostate cancer^35^ and additional changes have been made to the histopathological assessment.^38^ Both these changes could potentially again change the prostate cancer prognosis—especially in men with localised prostate cancer. It is unknown—but seems unlikely—that a prostate cancer found following an MRi targeted biopsy of a suspicious lesion has the same oncological risk as a similar prostate cancer found following systematic TRUS guided biopsies.^39^ It is essential that we continue to study the changes that new guidelines and modalities entails to understand how this affects our understanding of prostate cancer.

## 5. CONCLUSION

During the 11‐year study period, there was a gradual decrease in risk of death from nonprostate cancer causes in men with localised prostate cancer treated with radical prostatectomy in Sweden. A decrease in prostate cancer deaths was restricted to men with intermediate‐risk prostate cancer, likely explained by changes in disease characteristics most strongly affecting this risk category.

## CONFLICT OF INTERESTS

The authors declare that there are no conflict of interests.

## AUTHOR CONTRIBUTIONS

All authors contributed to the manuscript and approved of the final version. Frederik B. Thomsen had full access to the data in the study and takes responsibility for the integrity of the data and the accuracy of the data analysis. There were no other contributors to the manuscript except those on the author list.

## SYNOPSIS

In the period 2000–2010 there was a gradual decrease in risk of death from prostate cancer in men with localised intermediate‐risk prostate cancer treated with radical prostatectomy in Sweden.

## Supporting information

Supporting information.

Supporting information.

## Data Availability

The Research Ethics Board at Uppsala University approved of the linkages in our project (PCBaSe 2016‐239). We received a study file from Statistics Sweden and the National Board of Health and Welfare where the person identity number for men in the National prostate Cancer Register had been replaced by a code. This means that the data set is pseudononymized, but due to a large number of variables this data set is still considered not anonymized when deleting this code. The following restrictions apply: we are not allowed to share data on individuals with other researchers, nor or we allowed to upload such data on an open server. However, we can provide access to the data set on a remote server on demand. On the Research platform, data can be uploaded and then accessed by external researchers. However, no individual data are allowed to leave the platform but aggregated data in the form of figures and Tables can be exported. This study was approved by the Research Ethics Board at Uppsala University and included all men in NPCR who were diagnosed with localised prostate cancer in 2000–2010 and who underwent radical prostatectomy within 6 months from diagnosis. External researcher should contact the corresponding author who will direct the demand for data to the PCBaSe reference group who will then provider the access described above.
