# Supplementary material for: Temporal changes in cause‐specific death in men with localised prostate cancer treated with radical prostatectomy: a population‐based, nationwide study
Source: J Surg Oncol. 2021 Jun 18;124(5):867–75. doi: 10.1002/jso.26579 (PMC8518635; doi:10.1002/jso.26579)
Supplement: Supplementary file 1 — Supporting information. [file JSO-124-867-s001.docx]

| **Supplemental Table 1** Baseline characteristics of 19 330 men diagnosed with localised prostate cancer and treated with radical prostatectomy stratified on diagnostic period and risk category | | | | | | | | | | | | | | | | | |
| --- | --- | --- | --- | --- | --- | --- | --- | --- | --- | --- | --- | --- | --- | --- | --- | --- | --- |
|  | Low-risk | | | | |  | Intermediate-risk | | | | |  | High-risk | | | | |
|  | 2000-02 | 2003-04 | 2005-06 | 2007-08 | 2009-10 |  | 2000-02 | 2003-04 | 2005-06 | 2007-08 | 2009-10 |  | 2000-02 | 2003-04 | 2005-06 | 2007-08 | 2009-10 |
|  | n (%) | n (%) | n (%) | n (%) | n (%) |  | n (%) | n (%) | n (%) | n (%) | n (%) |  | n (%) | n (%) | n (%) | n (%) | n (%) |
| Age at diagnosis, years |  |  |  |  |  |  |  |  |  |  |  |  |  |  |  |  |  |
| <60 | 590  (39) | 740  (38) | 750  (35) | 613  (36) | 669  (36) |  | 315  (30) | 369  (25) | 440  (27) | 465  (26) | 550  (23) |  | 76  (29) | 91  (28) | 74  (21) | 88  (21) | 93  (17) |
| 60-<65 | 467  (31) | 631  (33) | 757  (35) | 590  (34) | 592  (32) |  | 340  (32) | 499  (34) | 552  (34) | 596  (34) | 743  (31) |  | 78  (29) | 100  (31) | 130  (37) | 137  (32) | 143  (27) |
| 65-<70 | 380  (25) | 452  (24) | 510  (24) | 413  (24) | 480  (26) |  | 308  (29) | 433  (30) | 442  (28) | 556  (31) | 778  (33) |  | 78  (29) | 88  (27) | 95  (27) | 146  (34) | 201  (38) |
| 70+ | 66  (4) | 100  (5) | 123  (6) | 103  (6) | 113  (6) |  | 91  (9) | 152  (10) | 173  (11) | 155  (9) | 289  (12) |  | 34  (13) | 45  (14) | 54  (15) | 55  (13) | 96  (18) |
| Clinical tumour category |  |  |  |  |  |  |  |  |  |  |  |  |  |  |  |  |  |
| T1 | 984  (65) | 1349  (70) | 1570  (73) | 1236  (72) | 1402  (76) |  | 542  (51) | 834  (57) | 977  (61) | 1101  (62) | 1509  (64) |  | 122  (46) | 176  (54) | 210  (59) | 223  (52) | 305  (57) |
| T2 | 519  (35) | 574  (30) | 570  (27) | 483  (28) | 452  (24) |  | 512  (49) | 619  (43) | 630  (39) | 671  (38) | 851  (36) |  | 144  (54) | 148  (46) | 143  (41) | 203  (48) | 228  (43) |
| Gleason score |  |  |  |  |  |  |  |  |  |  |  |  |  |  |  |  |  |
| ≤6 | 1474  (100) | 1917  (100) | 2137  (100) | 1719  (100) | 1854  100) |  | 436  (49) | 574  (40) | 483  (31) | 420  (24) | 426  (18) |  | 76  (32) | 96  (31) | 79  (23) | 85  (20) | 74  (14) |
| 7 (3+4) | 0  (0) | 0  (0) | 0  (0) | 0  (0) | 0  (0) |  | 334  (37) | 651  (46) | 776  (50) | 1004  (57) | 1394  (59) |  | 25  (11) | 32  (10) | 29  (9) | 51  (12) | 71  (13) |
| 7 (4+3) | 0  (0) | 0  (0) | 0  (0) | 0  (0) | 0  (0) |  | 125  (14) | 205  (14) | 302  (19) | 345  (20) | 538  (23) |  | 11  (5) | 16  (5) | 24  (7) | 35  (8) | 34  (6) |
| 8 | 0  (0) | 0  (0) | 0  (0) | 0  (0) | 0  (0) |  | 0  (0) | 0  (0) | 0  (0) | 0  (0) | 0  (0) |  | 97  (41) | 137  (44) | 167  (49) | 203  (48) | 276  (52) |
| 9-10 | 0  (0) | 0  (0) | 0  (0) | 0  (0) | 0  (0) |  | 0  (0) | 0  (0) | 0  (0) | 0  (0) | 0  (0) |  | 29  (12) | 31  (10) | 39  (12) | 51  (12) | 78  (15) |
| PSA at diagnosis, ng/mL |  |  |  |  |  |  |  |  |  |  |  |  |  |  |  |  |  |
| Median (IQR) | 6  (5-8) | 6  (5-8) | 6  (4-7) | 5  (4-7) | 5  (4-7) |  | 11  (8-14) | 10  (7-13) | 10  (6-12) | 8  (5-12) | 8  (5-11) |  | 20  (9-24) | 20  (9-25) | 16  (7-23) | 17  (8-24) | 12  (7-23) |
| Positive biopsy cores, % |  |  |  |  |  |  |  |  |  |  |  |  |  |  |  |  |  |
| Median (IQR) | 33  (17-50) | 33  (17-50) | 30  (17-40) | 30  (20-50) | 30  (18-42) |  | 33  (25-50) | 33  (25-50) | 33  (20-50) | 38  (25-50) | 38  (20-50) |  | 46  (32-50) | 33  (17-50) | 33  (17-50) | 33  (18-50) | 33  (20-50) |
| Charlson comorbidity index |  |  |  |  |  |  |  |  |  |  |  |  |  |  |  |  |  |
| 0 | 1382  (92) | 1736  (90) | 1902  (89) | 1519  (88) | 1627  (88) |  | 958  (91) | 1293  (89) | 1412  (88) | 1548  (87) | 2028  (86) |  | 244  (92) | 285  (88) | 298  (84) | 363  (85) | 444  (83) |
| 1 | 76  (5) | 134  (7) | 164  (8) | 145  (8) | 165  (9) |  | 73  (7) | 121  (8) | 144  (9) | 168  (9) | 233  (10) |  | 17  (6) | 28  (9) | 42  (12) | 46  (11) | 70  (13) |
| 2+ | 45  (3) | 53  (3) | 74  (3) | 55  (3) | 62  (3) |  | 23  (2) | 39  (3) | 51  (3) | 56  (3) | 99  (4) |  | 5  (2) | 11  (3) | 13  (4) | 17  (4) | 19  (4) |
| Abbreviation PSA prostate specific antigen; IQR interquartile range | | | | | | | | | | | | | | | | | |
