# Supplementary material for: Temporal changes in cause‐specific death in men with localised prostate cancer treated with radical prostatectomy: a population‐based, nationwide study
Source: J Surg Oncol. 2021 Jun 18;124(5):867–75. doi: 10.1002/jso.26579 (PMC8518635; doi:10.1002/jso.26579)
Supplement: Supplementary file 2 — Supporting information. [file JSO-124-867-s002.docx]

| **Supplemental** **Table 2** Multivariable Cox regression analyses for risk of death from prostate cancer stratified on risk category | | | | | | | | | | | | | | | | | | |
| --- | --- | --- | --- | --- | --- | --- | --- | --- | --- | --- | --- | --- | --- | --- | --- | --- | --- | --- |
|  |  | Low risk | | | | |  | Intermediate risk | | | | |  | High risk | | | | |
|  |  | Univariabel | |  | Multivariabel | |  | Univariabel | |  | Multivariabel | |  | Univariabel | |  | Multivariabel | |
|  |  | HR | 95% CI |  | HR | 95% CI |  | HR | 95% CI |  | HR | 95% CI |  | HR | 95% CI |  | HR | 95% CI |
| Diagnosis | |  |  |  |  |  |  |  |  |  |  |  |  |  |  |  |  |  |
|  | 2000-2002 | Ref |  |  | Ref |  |  | Ref |  |  | Ref |  |  | Ref |  |  | Ref |  |
|  | 2003-2004 | 0.61 | (0.32-1.16) |  | 0.66 | (0.30-1.42) |  | 0.79 | (0.57-1.10) |  | 1.10 | (0.72-1.69) |  | 0.84 | (0.66-1.07) |  | 1.36 | (0.83-2.24) |
|  | 2005-2006 | 0.64 | (0.33-1.27) |  | 0.70 | (0.31-1.58) |  | 0.67 | (0.47-0.95) |  | 0.86 | (0.55-1.35) |  | 0.65 | (0.50-0.85) |  | 0.79 | (0.45-1.39) |
|  | 2007-2008 | 0.63 | (0.28-1.42) |  | 0.74 | (0.30-1.83) |  | 0.42 | (0.28-0.64) |  | 0.56 | (0.33-0.95) |  | 0.66 | (0.49-0.87) |  | 0.98 | (0.56-1.69) |
|  | 2009-2010 | 0.52 | (0.20-1.34) |  | 0.54 | (0.20-1.50) |  | 0.36 | (0.23-0.57) |  | 0.47 | (0.27-0.82) |  | 0.59 | (0.43-0.80) |  | 0.80 | (0.45-1.42) |
| Age, years | |  |  |  |  |  |  |  |  |  |  |  |  |  |  |  |  |  |
|  | <60 | Ref |  |  | Ref |  |  | Ref |  |  | Ref |  |  | Ref |  |  | Ref |  |
|  | 60-<65 | 1.16 | (0.68-2.00) |  | 1.22 | (0.70-2.10) |  | 1.41 | (1.00-1.99) |  | 1.53 | (1.06-2.20) |  | 1.29 | (1.02-1.64) |  | 0.72 | (0.47-1.14) |
|  | 65-<70 | 1.91 | (1.13-3.23) |  | 1.82 | (1.06-3.11) |  | 1.84 | (1.31-2.57) |  | 1.93 | (1.35-2.76) |  | 1.90 | (1.51-2.40) |  | 0.94 | (0.61-1.45) |
|  | 70+ | 2.52 | (1.14-5.56) |  | 2.35 | (1.05-5.26) |  | 1.71 | (1.09-2.67) |  | 1.71 | (1.07-2.76) |  | 2.85 | (2.14-3.79) |  | 1.39 | (0.87-2.23) |
| Clinical tumour category | |  |  |  |  |  |  |  |  |  |  |  |  |  |  |  |  |  |
|  | T1 | Ref |  |  | Ref |  |  | Ref |  |  | Ref |  |  | Ref |  |  | Ref |  |
|  | T2 | 2.44 | (1.61-3.69) |  | 2.11 | (1.38-3.22) |  | 2.11 | (1.66-2.69) |  | 1.50 | (1.16-1.95) |  | 2.64 | (2.22-3.13) |  | 1.67 | (1.21-2.31) |
| Gleason score | |  |  |  |  |  |  |  |  |  |  |  |  |  |  |  |  |  |
|  | ≤6 | - |  |  | - |  |  | Ref |  |  | Ref |  |  | Ref |  |  | Ref |  |
|  | 7 (3+4) | - | - |  | - | - |  | 1.94 | (1.37-2.74) |  | 2.65 | (1.78-3.93) |  | 2.93 | (2.33-3.69) |  | 1.41 | (0.72-2.77) |
|  | 7 (4+3) | - | - |  | - | - |  | 3.46 | (2.38-5.04) |  | 3.02 | (3.02-6.93) |  | 5.28 | (4.06-6.86) |  | 2.20 | (1.07-4.51) |
|  | 8 | - | - |  | - | - |  | - | - |  | - | - |  | 7.67 | (5.79-10.16) |  | 1.67 | (0.79-3.54) |
|  | 9-10 | - | - |  | - | - |  | - | - |  | - | - |  | 25.03 | (18.27-34.25) | | 4.78 | (2.22-10.28) |
| PSA, ng/mL | |  |  |  |  |  |  |  |  |  |  |  |  |  |  |  |  |  |
|  | 0-10 | - |  |  | - |  |  | Ref |  |  | Ref |  |  | Ref |  |  | Ref |  |
|  | 10-20 | - | - |  | - | - |  | 1.10 | (0.86-1.39) |  | 1.63 | (1.23-2.17) |  | 2.09 | (1.74-2.51) |  | 0.83 | (0.56-1.23) |
|  | 20-50 | - | - |  | - | - |  | - | - |  | - | - |  | 3.48 | (2.64-4.57) |  | 0.79 | (0.42-1.47) |
| PPB | |  |  |  |  |  |  |  |  |  |  |  |  |  |  |  |  |  |
|  | <16.7% | Ref |  |  | Ref |  |  | Ref |  |  | Ref |  |  | Ref |  |  | Ref |  |
|  | 16.7-33% | 0.95 | (0.35-2.63) |  | 1.00 | (0.36-2.78) |  | 1.35 | (0.70-2.60) |  | 1.61 | (0.81-3.19) |  | 1.34 | (0.67-2.69) |  | 1.40 | (0.68-2.87) |
|  | 34-50% | 2.46 | (1.21-4.99) |  | 2.18 | (1.07-4.44) |  | 2.61 | (1.55-4.40) |  | 2.43 | (1.39-4.22) |  | 2.61 | (1.53-4.47) |  | 2.22 | (1.27-3.89) |
|  | >50% | 4.08 | (1.88-8.84) |  | 3.35 | (1.52-7.39) |  | 4.55 | (2.70-7.68) |  | 4.07 | (2.33-7.12) |  | 4.02 | (2.34-6.92) |  | 3.53 | (1.98-6.30) |
|  | Missing | 2.61 | (1.23-5.54) |  | 1.73 | (0.76-3.95) |  | 4.26 | (2.49-7.27) |  | 3.05 | (1.63-5.71) |  | 2.43 | (1.31-4.46) |  | 2.34 | (1.17-1.67) |
| CCI | |  |  |  |  |  |  |  |  |  |  |  |  |  |  |  |  |  |
|  | 0 | Ref |  |  | Ref |  |  | Ref |  |  | Ref |  |  | Ref |  |  | Ref |  |
|  | 1 | 1.16 | (0.44-2.30) |  | 1.02 | (0.44-2.35) |  | 1.09 | (0.71-1.65) |  | 0.98 | (0.62-1.55) |  | 1.16 | (0.86-1.56) |  | 1.00 | (0.60-1.67) |
|  | 2+ | 1.93 | (0.69-5.12) |  | 1.77 | (0.64-4.87) |  | 0.88 | (0.39-1.97) |  | 0.91 | (0.40-2.04) |  | 1.93 | (1.29-2.88) |  | 4.16 | (2.33-7.43) |
| Education | |  |  |  |  |  |  |  |  |  |  |  |  |  |  |  |  |  |
|  | Low | Ref |  |  | Ref |  |  | Ref |  |  | Ref |  |  | Ref |  |  | Ref |  |
|  | Middle | 1.00 | (0.51-1.38) |  | 0.99 | (0.60-1.64) |  | 0.85 | (0.64-1.12) |  | 0.93 | (0.69-1.25) |  | 0.81 | (0.67-0.99) |  | 0.85 | (0.59-1.22) |
|  | High | 1.88 | (0.52-1.51) |  | 1.14 | (0.66-1.96) |  | 0.80 | (0.59-1.09) |  | 0.89 | (0.64-1.24) |  | 0.79 | (0.64-0.99) |  | 0.91 | (0.61-1.34) |
|  | Missing | - | - |  | - | - |  | 0.74 | (0.10-5.23) |  | 0.71 | (0.10-5.11) |  | 1.41 | (0.45-4.42) |  | 1.20 | (0.16-8.92) |
| Marital status | |  |  |  |  |  |  |  |  |  |  |  |  |  |  |  |  |  |
|  | Married | Ref |  |  | Ref |  |  | Ref |  |  | Ref |  |  | Ref |  |  | Ref |  |
|  | Not married | 1.67 | (1.08-2.57) |  | 1.88 | (1.21-2.93) |  | 0.91 | (0.69-1.20) |  | 0.94 | (0.70-1.26) |  | 1.11 | (0.91-1.37) |  | 1.17 | (0.83-1.66) |
| No. procedures performed at treating hospital | |  |  |  |  |  |  |  |  |  |  |  |  |  |  |  |  |  |
|  | 1st Quartile | Ref |  |  | Ref |  |  | Ref |  |  | Ref |  |  | Ref |  |  | Ref |  |
|  | 2nd Quartile | 0.83 | (0.52-1.33) |  | 0.58 | (0.58-1.55) |  | 0.90 | (0.68-1.18) |  | 1.08 | (0.80-1.45) |  | 1.03 | (0.91-1.95) |  | 1.26 | (0.85-1.87) |
|  | 3rd Quartile | 1.23 | (0.54-2.78) |  | 1.46 | (0.64-3.34) |  | 1.24 | (0.69-2.22) |  | 1.16 | (0.57-2.33) |  | 1.48 | (0.74-2.99) |  | 1.22 | (0.59-2.54) |
|  | 4th Quartile | 0.41 | (0.20-0.81) |  | 0.48 | (0.24-0.99) |  | 0.64 | (0.45-0.91) |  | 0.83 | (0.56-1.23) |  | 1.02 | (0.63-1.64) |  | 0.90 | (0.55-1.49) |
| Abbreviation PSA prostate specific-antigen, PPB percent positive biopsy cores, CCI Charlson comorbidity index | | | | | | | | | | | | | | | | | | |
